# Pim Kinase Inhibitors Increase Gilteritinib Cytotoxicity in FLT3-ITD Acute Myeloid Leukemia Through GSK-3β Activation and c-Myc and Mcl-1 Proteasomal Degradation

**DOI:** 10.1158/2767-9764.CRC-23-0379

**Published:** 2024-02-16

**Authors:** Jonelle K. Lee, Aditi Chatterjee, Mario Scarpa, Christopher M. Bailey, Sandrine Niyongere, Prerna Singh, Moaath K. Mustafa Ali, Shivani Kapoor, Yin Wang, Giovannino Silvestri, Maria R. Baer

**Affiliations:** 1 University of Maryland Greenebaum Comprehensive Cancer Center, Baltimore, Maryland.; 2 Department of Medicine, University of Maryland School of Medicine, Baltimore, Maryland.; 3 Institute of Human Virology, University of Maryland School of Medicine, Baltimore, Maryland.; 4 Veterans Affairs Medical Center, Baltimore, Maryland.

## Abstract

Acute myeloid leukemia (AML) with *fms-*like tyrosine kinase 3 internal tandem duplication (FLT3-ITD) has poor outcomes. FLT3-ITD drives constitutive and aberrant FLT3 signaling, activating STAT5 and upregulating the downstream oncogenic serine/threonine kinase Pim-1. FLT3 inhibitors are in clinical use, but with limited and transient efficacy. We previously showed that concurrent treatment with Pim and FLT3 inhibitors increases apoptosis induction in FLT3-ITD–expressing cells through posttranslational downregulation of Mcl-1. Here we further elucidate the mechanism of action of this dual targeting strategy. Cytotoxicity, apoptosis and protein expression and turnover were measured in FLT3-ITD–expressing cell lines and AML patient blasts treated with the FLT3 inhibitor gilteritinib and/or the Pim inhibitors AZD1208 or TP-3654. Pim inhibitor and gilteritinib cotreatment increased apoptosis induction, produced synergistic cytotoxicity, downregulated c-Myc protein expression, earlier than Mcl-1, increased turnover of both proteins, which was rescued by proteasome inhibition, and increased efficacy and prolonged survival in an *in vivo* model. Gilteritinib and Pim inhibitor cotreatment of Ba/F3-ITD cells infected with T58A c-Myc or S159A Mcl-1 plasmids, preventing phosphorylation at these sites, did not downregulate these proteins, increase their turnover or increase apoptosis induction. Moreover, concurrent treatment with gilteritinib and Pim inhibitors dephosphorylated (activated) the serine/threonine kinase glycogen synthase kinase-3β (GSK-3β), and GSK-3β inhibition prevented c-Myc and Mcl-1 downregulation and decreased apoptosis induction. The data are consistent with c-Myc T58 and Mcl-1 S159 phosphorylation by activated GSK-3β as the mechanism of action of gilteritinib and Pim inhibitor combination treatment, further supporting GSK-3β activation as a therapeutic strategy in FLT3-ITD AML.

**Significance:**

FLT3-ITD is present in 25% of in AML, with continued poor outcomes. Combining Pim kinase inhibitors with the FDA-approved FLT3 inhibitor gilteritinib increases cytotoxicity *in vitro* and *in vivo* through activation of GSK-3β, which phosphorylates and posttranslationally downregulates c-Myc and Mcl-1. The data support efficacy of GSK-3β activation in FLT3-ITD AML, and also support development of a clinical trial combining the Pim inhibitor TP-3654 with gilteritinib.

## Introduction

Internal tandem duplication of the *fms*-like tyrosine kinase 3 receptor tyrosine kinase (FLT3-ITD) is present in acute myeloid leukemia (AML) in 30% of patients (1). These patients respond to chemotherapy but relapse rapidly and have poor outcomes (2). While outcomes have improved with incorporation of FLT3 inhibitors and allogeneic hematopoietic stem cell transplantation into treatment (3), the efficacy of FLT3 inhibitors is limited by incomplete responses and onset of resistance, and may be enhanced by dual targeting of FLT3-ITD signaling pathways (4).

FLT3-ITD drives constitutive and aberrant FLT3 signaling. The wild-type FLT3 receptor signals through the PI3K-AKT-mTOR and Ras-Raf-MEK-ERK pathways upon binding of FLT3 ligand (5). FLT3-ITD additionally aberrantly activates signal transducer and activator of transcription (STAT) 5, upregulating the downstream oncogenic serine/threonine kinase proviral integration site for Moloney murine leukemia virus 1 (Pim-1; refs. 6, 7), the c-Myc oncogene (7) and the antiapoptotic protein Mcl-1 (8). Pim-1, one of three Pim kinase isoforms, Pim-1, Pim-2, and Pim-3, not only contributes directly to the proliferative and antiapoptotic effects of FLT3-ITD, but also phosphorylates and stabilizes FLT3 in a positive feedback loop in cells with FLT3-ITD (9, 10). Dual inhibition of Pim and FLT3 kinases has been shown to enhance cytotoxicity and apoptosis induction in cell lines and primary AML cells with FLT3-ITD (9–15). Pim kinase inhibitors are in clinical development (16, 17) and combining Pim and FLT3 inhibitors is a promising treatment strategy for AML with FLT3-ITD.

c-Myc is a transcription factor that dimerizes with its coactivator, Max, to induce expression of a number of gene families, driving both proliferation and resistance to apoptosis induction (18). In addition to being transcriptionally upregulated in AML with FLT3-ITD (7), c-Myc is also regulated posttranslationally by Pim-1 (19) and by the serine/threonine kinase glycogen synthase kinase-3β (GSK-3β; ref. 20). Pim-1–mediated phosphorylation of c-Myc at S62 and decreased phosphorylation at T58 result in posttranslational upregulation via stabilization (19), while GSK-3β phosphorylation of c-Myc at T58 promotes its proteasomal degradation (20). GSK-3β, in turn, is a substrate of both AKT (21) and Pim-1 kinase (22), both of which phosphorylate it at S9, thereby rendering it catalytically inactive (21, 23). In addition, we showed that GSK-3β also phosphorylates Pim-1, resulting in its posttranslational downregulation, creating a negative feedback loop between these two kinases (24).

We previously showed that concurrent Pim and FLT3 inhibition increases apoptosis induction in cells with FLT3-ITD, but not wild-type FLT3, through posttranslational downregulation of the antiapoptotic protein Mcl-1 (12), which is also upregulated in AML with FLT3-ITD (8). Mcl-1 is also phosphorylated by GSK-3β, at S159, and thereby tagged for degradation (25). Here we demonstrate that Pim and FLT3 inhibitor cotreatment activates GSK-3β in cells with FLT3-ITD, resulting in posttranslational downregulation of both c-Myc and Mcl-1.

## Materials and Methods

### Cell Lines

Cell lines studied included Ba/F3-ITD (Ba/F3 murine pro-B cells stably transfected with human FLT3-ITD, from Dr. Mark Levis, Johns Hopkins University School of Medicine, Baltimore, MD) and MV4-11 (ATCC, #CRL-9591; RRID:CVCL_0064) and MOLM-14 (ATCC, #CRL-9589; RRID:CVCL_7916) human AML cells, with homozygous and heterozygous FLT3-ITD, respectively. Cell lines were cultured as described previously (12). They were tested for *Mycoplasma* every 6 months with the MycoAlert Mycoplasma Detection Kit (Lonza, #LT07-118); most recent testing, for the work here, was on February 14, 2023.

### Retroviral and Lentiviral Infection of Ba/F3-ITD Cells

Retroviral packaging and infection of Ba/F3-ITD cells were performed as described previously (24). For lentiviral infection, lentiviral particles were generated through transient calcium phosphate transfection using the ProFection Mammalian Transfection System (Promega, #E1200), applied to HEK293T cells (ATCC, #CRL-3216; RRID:CVCL_0063). The viral supernatant, collected 24 and 48 hours posttransfection, was concentrated through polyethylene glycol (Research Products International, #P48080) precipitation. Ba/F3-ITD cell line infections underwent three rounds of spinoculation. Polybrene (Millipore Sigma, #TR1003-G; 4 µg/mL) was added to viral supernatants, and puromycin selection was initiated 48 hours after transduction.

Ba/F3-ITD cells were subjected to lentiviral infection with pCDH-MSCV-MycT58A-EF1a-copGFP plasmid containing c-Myc with a mutation changing threonine to alanine at residue 58, preventing phosphorylation, or pCDH-MSCV-Myc(WT)-EF1a-copGFP plasmid containing wild-type c-Myc, or pCDH-MSCV-MCS-EF1-copGFP empty vector control, generous gifts from Dr. Timothy Phoenix, Division of Pharmaceutical Sciences, James L. Winkle College of Pharmacy, University of Cincinnati, Cincinnati, OH (26).

Ba/F3-ITD cells also underwent retroviral infection with pBabe-Flag hMcl-1-S159A plasmid (Addgene plasmid # 25372; RRID:Addgene_25372) containing Mcl-1 with a mutation changing serine to alanine at residue 159, preventing phosphorylation (27), pBabe-Flag hMcl-1 plasmid (Addgene plasmid # 25371; RRID:Addgene_25371), containing wild-type Mcl-1 (27), or pBabe-puro empty vector control (Addgene plasmid # 1764; RRID:Addgene_1764; ref. 24).

Finally, Ba/F3-ITD cells underwent retroviral infection with pBabe-Puro-Myr-Flag-AKT1 (Addgene plasmid # 15294; RRID:Addgene_15294; ref. 28), containing myristoylated (constitutively activated) AKT (myr-AKT), or pBABE-puro empty vector control, as described previously (24).

### Patient Samples

Blood samples were obtained from patients with AML with FLT3-ITD with peripheral blasts on a University of Maryland School of Medicine Institutional Review Board–approved tissue procurement protocol, following written informed consent. Studies were conducted in accordance with the Declaration of Helsinki. Peripheral blood mononuclear cells were isolated by density centrifugation over Ficoll-Paque (Millipore Sigma, #GE17-1440-02). Patient sample clinical information is summarized in Supplementary Table S1.

### Materials

The FLT3 inhibitor gilteritinib (ASP2215; MedChemExpress, #HY-12432) was used at clinically relevant (29) concentrations of 10 or 15 nmol/L in *in vitro* experiments, consistent with concentrations that inhibited FLT3 and STAT5 inhibition in cells with FLT3-ITD (30, 31) and IC_50_ concentrations in cells with FLT3-ITD (24, 30, 31) and concentrations used *in vitro* in prior literature (24, 30, 31). The pan-Pim inhibitor AZD1208 (Tocris Bioscience, #6310) and Pim-1 inhibitor TP-3654 (MedChemExpress, #HY-101126) were both used at 1 µmol/L based on inhibition of p-BAD (S112) at this concentration (32, 33). The GSK-3β inhibitor TC-G 24 (Tocris, #4353) was used at 17 nmol/L based on GSK-3β inhibition (24).

### Measurement of Apoptosis

Cells were stained with Annexin V (BD Biosciences, #556419) and propidium iodide (PI; BD Biosciences, #556463) and analyzed on a FACS Canto II flow cytometer (BD Biosciences) using FlowJo v10.9 software (BD Biosciences; RRID:SCR_008520), as described previously (12).

### Drug Combination Studies

Cells were seeded in a 96-well plate and treated in triplicate with gilteritinib and Pim inhibitor at various concentrations alone and in combinations. WST-1 Cell Proliferation Reagent (Millipore Sigma, #11644807001) was added after 48 hours to terminate the assay. Drug combination effects were determined using the Chou-Talalay method, analyzed with CompuSyn software (ComboSyn; RRID:SCR 022931; ref. 34).

### *In Vivo* Study

Exponentially growing MV4-11-luc cells (1 × 10^6^; refs. 12, 24) were injected intravenously into the lateral tail veins of restrained female NOD-Rag1null IL2rgnull, NOD rag gamma (NRG) mice (6–8 weeks old). Cell engraftment was assessed 7 days later on a Xenogen IVIS Imaging System (Revvity, #124262) after intraperitoneal injection of 150 mg/kg d-luciferin (Millipore Sigma, #50227). Mice were sorted into four treatment groups, 5 mice in each, with equal mean signal intensity.

In a first experiment, treatment was initiated with gilteritinib 7.5 mg/kg and/or TP-3654 50 mg/kg in 5% DMSO, 40% polyethylene glycol 300 (PEG 300), 5% polysorbate 80 (Tween 80) and 50% water, or vehicle control, all by oral gavage, once every other day for three doses, followed by 2 days rest, each week. Gilteritinib dose and schedule were chosen to enhance demonstration of synergy.

In a second experiment, mice were treated with gilteritinib 15 mg/kg and/or TP-3654 50 mg/kg, or vehicle control, 5 days per week each week. Gilteritinib administered at 10 mg/kg orally daily was previously shown to effectively suppress FLT3 and STAT5 phosphorylation in an MV4-11 mouse model, leading to sustained antitumor activity (35). Gilteritinib reaches its maximum efficacy in patients at 80 mg or orally per day (29), with an approved clinical dose of 120 mg orally daily. Gilteritinib 15 mg/kg has been used previously in combination experiments *in vivo* (36).

Mice were weighed prior to each treatment. Leukemia burden was assessed weekly by noninvasive luciferin imaging, as above. Bioluminescent image data were analyzed with Living Image software (Revvity, #128113). Endpoints were 20% body weight loss, hind limb paralysis or lack of mobility to eat/drink. The University of Maryland Institutional Animal Care and Use Committee approved the study.

### Immunoblotting

Cells were lysed in 150 mmol/L NaCl lysis buffer with protease and phosphatase inhibitors (Millipore Sigma, #4906845001). Proteins were quantified using the Pierce bicinchoninic acid Protein Assay Kit (Thermo Fisher Scientific, #23225) and 15 µg (cell lines) or 50–75 µg (primary AML cells) from each sample were immunoblotted. Immunoblots were incubated with Cell Signaling Technology polyclonal primary antibodies to c-Myc (#9402; RRID:AB_2151827) or Mcl-1 (#4572; RRID:AB_2281980) or monoclonal primary antibodies to p-GSK-3α/β (S9/S21; 8566, RRID:AB_10860069), GSK-3α/β (#5676; RRID:AB_10547140), p-GSK-3β (S9; #9322; RRID:AB_2115196), GSK-3β (#9832; RRID:AB_10839406), p-AKT (S473; #4060; RRID: AB_2315049), p-AKT (T308; #2965; RRID:AB_2255933), AKT (#9272; RRID:AB_329827), p-p44/42 MAPK (ERK1/2; T202/Y204; #9101; RRID:AB_331646), p44/42MAPK (ERK1/2; #9107; RRID:AB_10695739) or β-actin (#3700; RRID:AB_2242334) or Sigma-Aldrich polyclonal primary antibody tpvinculin (#V9264; RRID:AB_10603627) overnight at 4°C, then with horseradish peroxidase–conjugated secondary antibodies for 1 hour at room temperature. Band intensities measured by densitometry (VisionWorks LS, UVP) at serial timepoints and normalized to vinculin controls were compared with intensity at time 0, defined as 100%.

### Protein Turnover and Proteasomal Degradation

To study protein turnover, cells were treated with 100 µg/mL cycloheximide (CHX; Millipore Sigma, #C7698) for 60 minutes to block new protein translation before adding gilteritinib and/or Pim inhibitor, or DMSO control. To study the effect of proteasomal degradation, cells were treated with CHX as above, with or without addition of the proteasome inhibitor carbobenzoxy-L-leucyl-L-leucyl-L-leucinal (MG-132;Millipore Sigma, #474790; 20 µmol/L) 30 minutes after adding CHX and 30 minutes before gilteritinib and/or Pim inhibitor, or DMSO control, treatment. Protein expression was measured at serial timepoints by immunoblotting. Band intensities were measured by densitometry, as above, and 50% protein turnover timepoints were determined using the line of best fit.

### Statistical Analysis

Flow cytometric analysis was performed on data from at least three independent experiments. Datapoints were pooled, with error bars representing SEM. Statistical analysis was performed by unpaired *t* test, using GraphPad Prism 9 (GraphPad Prism, GraphPad Software, RRID:SCR_002798). In the *in vivo* model, photon intensity in mice treated with TP-3654 and gilteritinib combination versus gilteritinib alone was compared by two-way ANOVA with Sidak multiple comparison test, and survival was compared by Kaplan–Meier analysis. Immunoblotting experiments were performed at least twice. Single immunoblots and corresponding densitometric analysis are shown in figures.

### Data Availability

The data generated in this study are available within the article.

## Results

### FLT3 and Pim Inhibitor Cotreatment is Synergistic in Cells with FLT3-ITD

**FIGURE 1:**
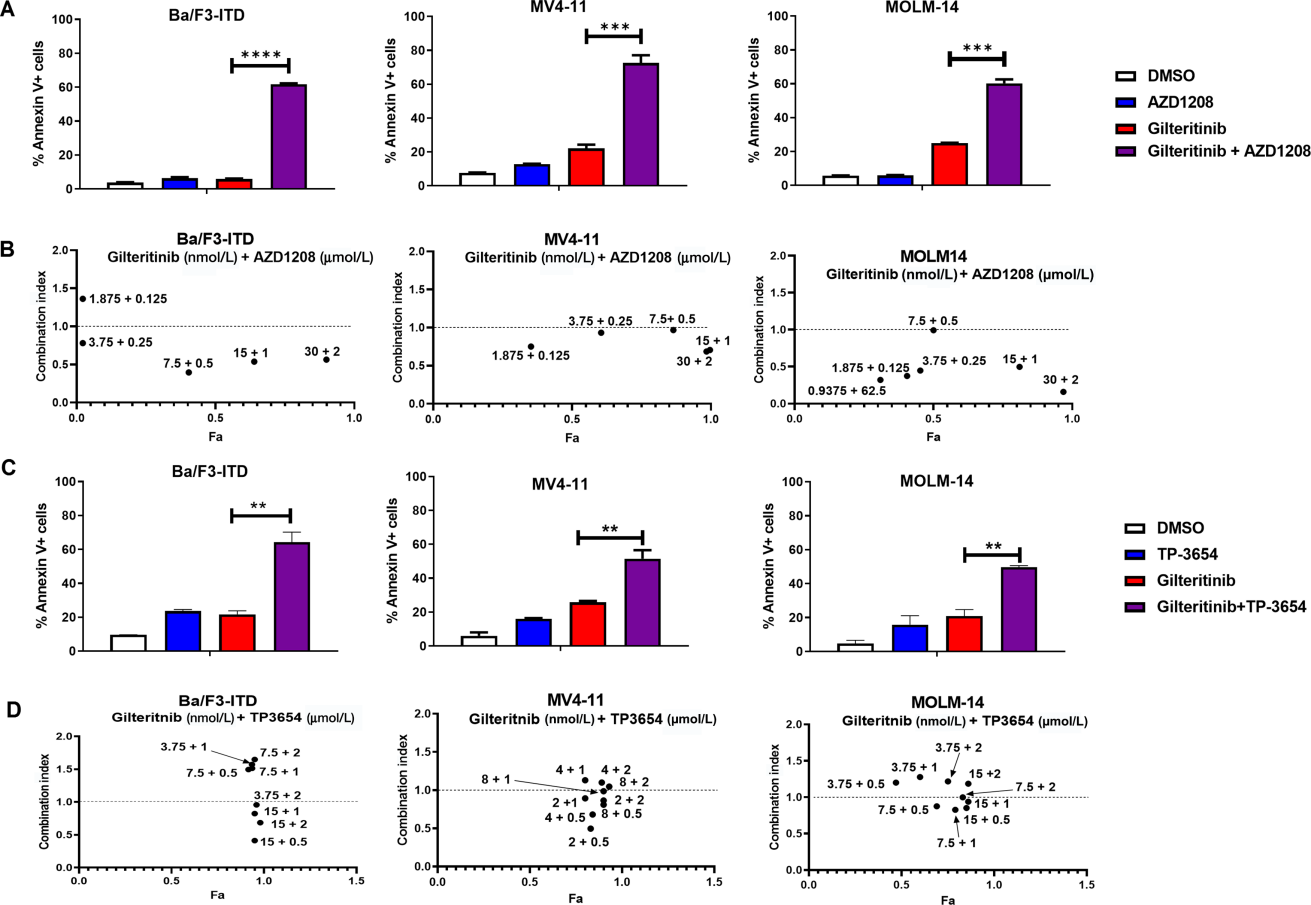
Pim inhibitor and gilteritinib combination treatment is synergistic in cells with FLT3-ITD. **A,C,** Apoptosis induction. Ba/F3-ITD, MV4-11, and MOLM-14 cells were treated with the FLT3 inhibitor gilteritinib (15 nmol/L for Ba/F3-ITD and 10 nmol/L for MV4-11 and MOLM-14) and/or the Pim inhibitor AZD1208 (1 µmol/L; A) or TP-3654 (1 µmol/L; C) or DMSO control for 48 hours in triplicate experiments**.** Apoptosis was analyzed by Annexin V and PI staining, measured by flow cytometry. ****, *P* < 0.0001; ***, *P* < 0.001; **, *P* < 0.01. **B,D,** Cytotoxicity. Ba/F3-ITD cells seeded at 5,000 cells/well and MV4-11 and MOLM-14 cells at 10,000 cells/well in 96-well plates were treated for 48 hours with gilteritinib and/or AZD1208 (B) or TP-3654 (D) as single drugs and in combinations at the concentrations shown, in triplicate. Cytotoxicity was measured by the WST-1 assay, and drug combination effects were determined by Chou-Talalay analysis. Synergism was defined by combination index values < 0.8.

We previously showed that cotreatment with the pan-Pim inhibitor AZD1208 and the FLT3 inhibitor quizartinib enhanced cytotoxicity, compared with single drugs, in cells with FLT3-ITD, but not wild-type FLT3, *in vitro* and *in vivo* (12). Here we studied efficacy of the FDA-approved FLT3 inhibitor gilteritinib and AZD1208 or the novel pan-Pim-1 inhibitor TP-3654, currently in clinical trials (refs. 37, 38; Fig. 1). Ba/F3-ITD, MV4-11 and MOLM-14 cells were treated with gilteritinib and/or AZD1208 (Fig. 1A; Supplementary Fig. S1) or TP-S3654 (Fig. 1C; Supplementary Fig. S1), or DMSO control, and apoptosis was measured by Annexin V/PI staining. Both combinations significantly increased apoptosis induction, compared with gilteritinib alone, in all three cell lines. Ba/F3-ITD, MV4-11, and MOLM-14 cells were also treated with gilteritinib and AZD1208 (Fig. 1B) or TP-3654 (Fig. 1D) as single drugs and combinations at different concentrations. Synergistic effects of both drug combinations were demonstrated by Chou-Talalay analysis in all three cell lines.

### *In Vivo* Efficacy of TP-3654 and Gilteritinib

**FIGURE 2:**
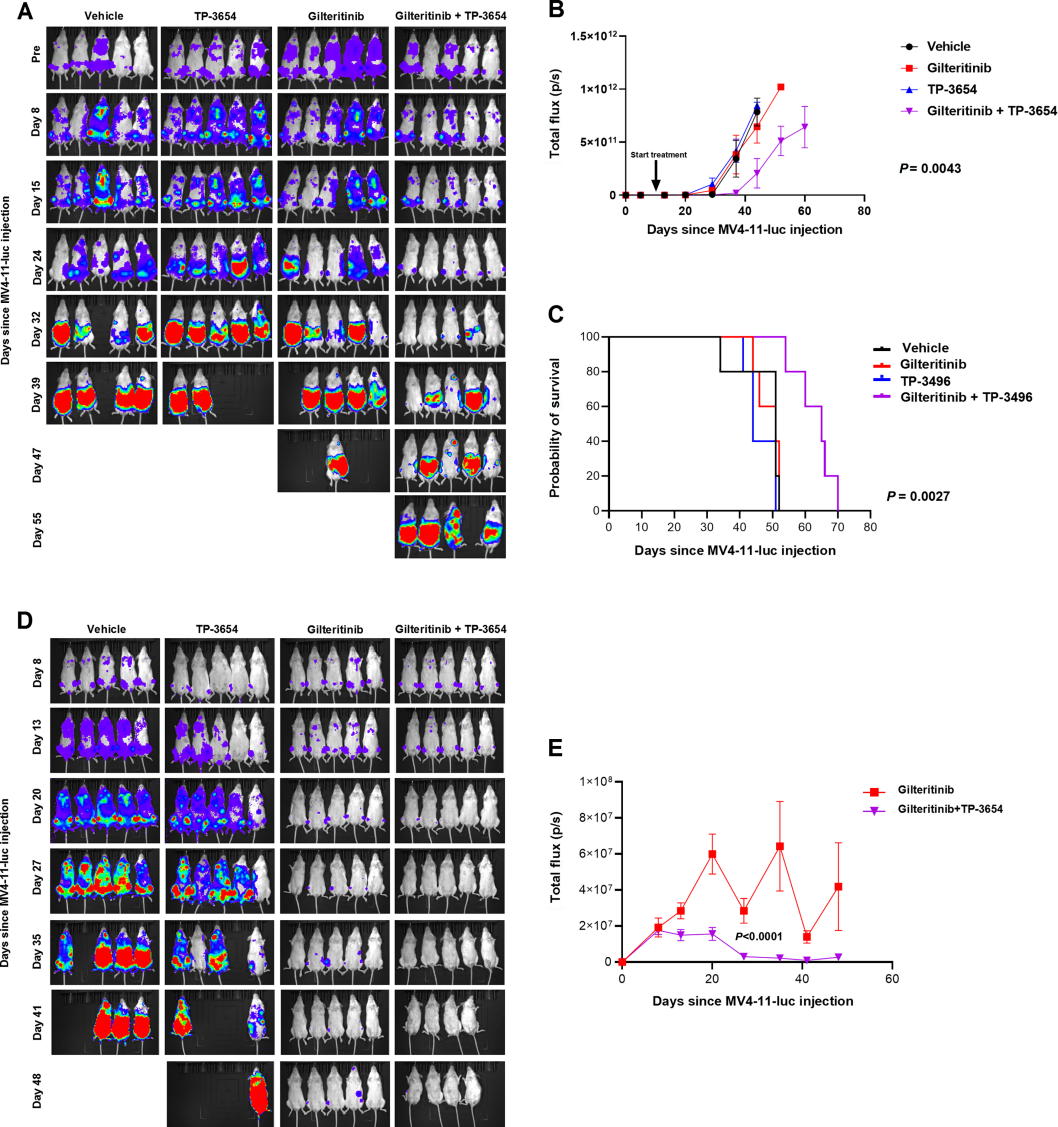
TP-3654 enhances efficacy of gilteritinib *in vivo*. NRG mice injected intravenously with MV4-11-luc cells were treated with gilteritinib and/or TP-3654, or vehicle control, with first treatment day defined as day 1. **A,** Serial images of mice in first experiment, treated with gilteritinib 7.5 mg/kg and/or TP-3654 50 mg/kg, or vehicle control, once every other day for three doses, followed by 2 days rest, each week. **B,** Changes in photon intensity, measured by bioluminescence imaging, over time, with *P* = 0.0043, comparing gilteritinib and TP-3654 combination versus gilteritinib alone on day 60 by two-way ANOVA with Sidak multiple comparison test. **C,** Survival curves, with *P* = 0.0027 by Kaplan–Meier analysis. Median survival was 51, 44, 51, and 65 days for mice treated with vehicle, TP-3654, gilteritinib, and gilteritinib and TP-3654 combination, respectively. **D,** Serial images of mice in second experiment, treated with gilteritinib 15 mg/kg and/or TP-3654 50 mg/kg, or vehicle control, daily for 5 days each week. **E,** Change in photon intensity, measured by bioluminescence imaging, over time, with *P* < 0.0001, comparing gilteritinib and TP-3654 combination versus gilteritinib alone by day 35 by two-way ANOVA.

In the *in vivo* model (Fig. 2), luminescence decreased significantly over time in mice engrafted with MV4-11-luc cells treated with TP-3654 and gilteritinib, compared with gilteritinib alone (Fig. 2A, B, D, and E), and treatment with TP-3654 and gilteritinib combination significantly prolonged survival (Fig. 2C).

### FLT3 and Pim Inhibitor Cotreatment Downregulates c-Myc Protein Expression, Earlier Than Mcl-1

**FIGURE 3:**
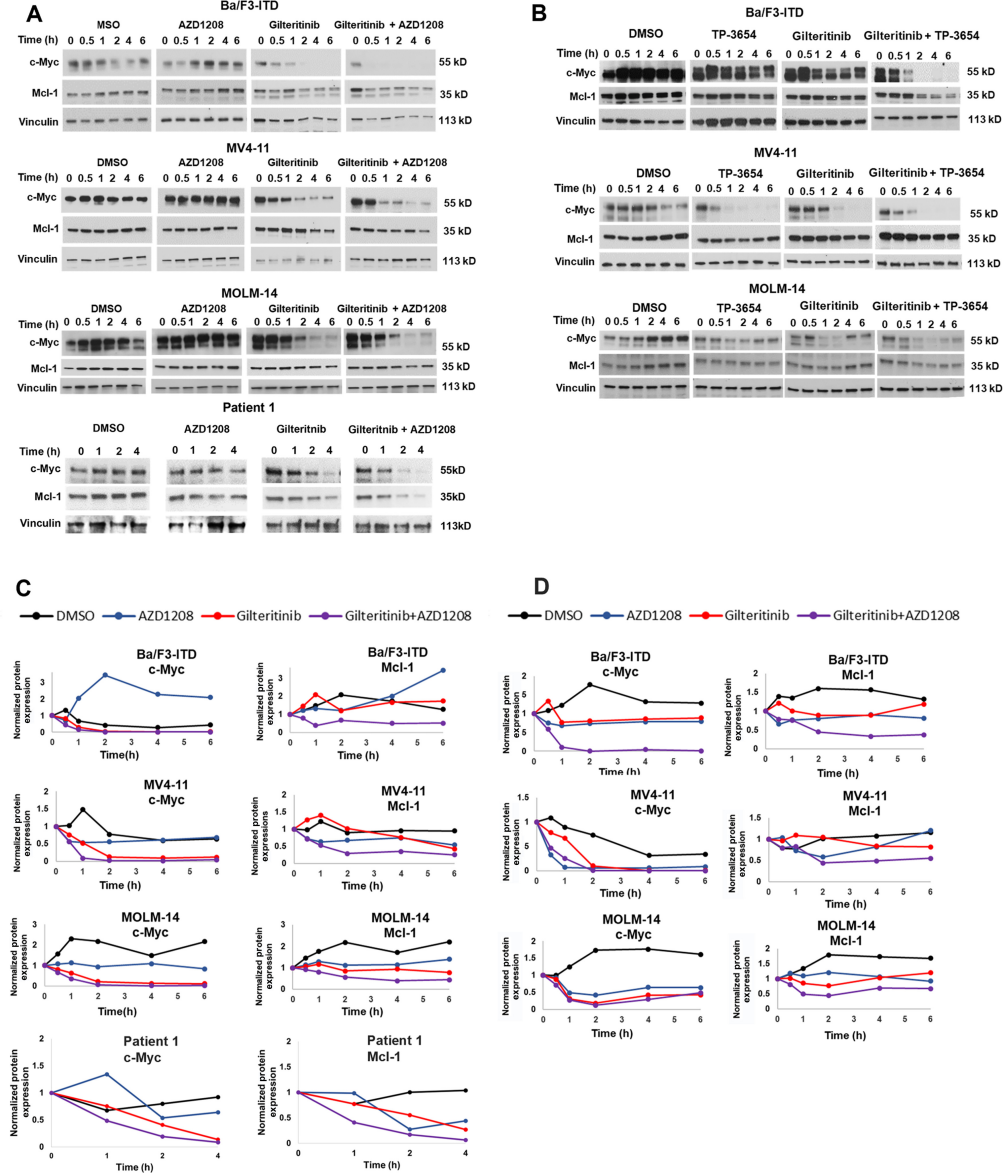
Concurrent Pim and FLT3 inhibition downregulates c-Myc prior to Mcl-1 protein expression. **A,** Ba/F3-ITD, MV4-11 and MOLM-14 cells and blasts from a patient with FLT3-ITD AML plated at 1 × 10^5^ cells/mL were treated with gilteritinib (15 nmol/L for Ba/F3-ITD and patient blasts and 10 nmol/L for MV4-11 and MOLM-14) and/or 1 µmol/L AZD1208, or DMSO control. **B,** Ba/F3-ITD, MV4-11, and MOLM-14 cells were also treated with gilteritinib and/or 1 µmol/L TP-3654, or DMSO control. c-Myc, Mcl-1, and vinculin loading control protein levels were measured by immunoblotting in samples collected at serial timepoints. Data in A and B are shown graphically in **C** and **D**, respectively.

To determine the effects of gilteritinib and Pim inhibitor cotreatment on expression of c-Myc, relative to Mcl-1, Ba/F3-ITD, MV4-11, and MOLM-14 cells and blasts from a patient with AML with FLT3-ITD were treated with gilteritinib and/or AZD1208, or DMSO control, and Ba/F3-ITD, MV4-11, and MOLM-14 cells were also treated with gilteritinib and/or TP-3654, or DMSO control. Samples collected at serial timepoints were analyzed by immunoblotting. c-Myc was downregulated to a greater extent by combination treatment than by gilteritinib alone, as was Mcl-1, and c-Myc downregulation preceded Mcl-1 downregulation (Fig. 3).

### Pim and FLT3 Inhibitor Cotreatment Downregulates c-Myc and Mcl-1 Protein Posttranslationally, Through Increased Proteasomal Degradation

**FIGURE 4:**
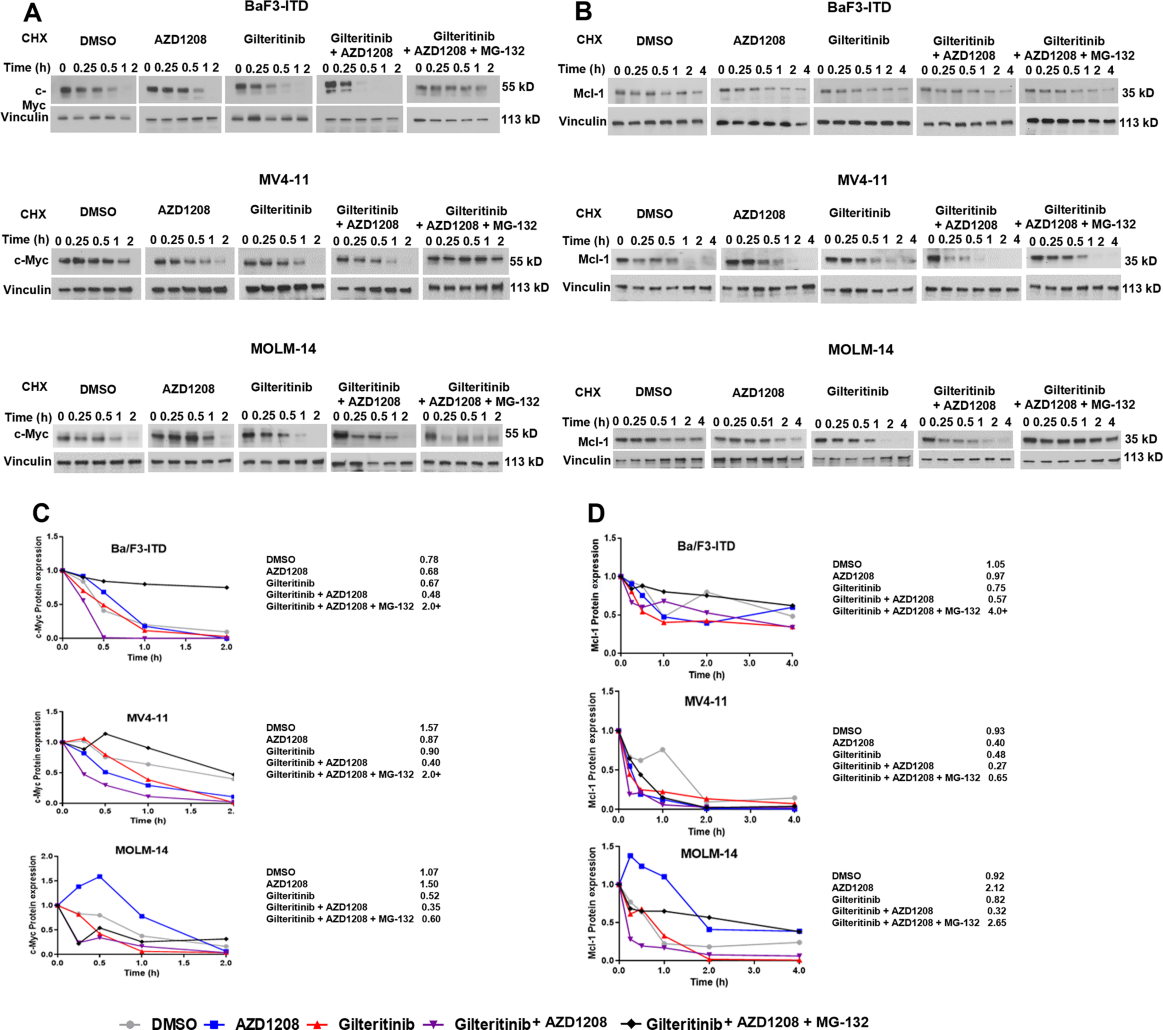
c-Myc and Mcl-1 are downregulated through enhanced proteasomal degradation. **A** and **B,** Ba/F3-ITD, MV4-11, and MOLM-14 cells plated at 1 × 10^5^ cells/mL were treated with 100 µg/mL CHX for 1 hour to inhibit protein synthesis, with or without addition of the proteasome inhibitor MG-132 after 30 minutes, prior to treatment with gilteritinib and/or AZD1208 at same concentrations as in Fig. 3, or DMSO control. Samples collected at serial timepoints were studied for expression of c-Myc (A), Mcl-1 (B) and vinculin loading control protein by immunoblotting. Data in A and B are shown graphically and numerically in **C** and **D**, respectively.

To determine the mechanism(s) by which gilteritinib and Pim inhibitor cotreatment downregulates c-Myc, Ba/F3-ITD, MV4-11, and MOLM-14 cells were pretreated for 1 hour with CHX with or without addition of MG-132 after 30 minutes, then treated with gilteritinib and/or AZD1208, or DMSO control (Fig. 4). c-Myc protein was measured by immunoblotting at serial timepoints, with vinculin loading control (Fig. 4A). AZD1208 and gilteritinib cotreatment accelerated c-Myc protein turnover, relative to single drugs or DMSO control, in all three cell lines, and accelerated turnover was abrogated by MG-132 pretreatment (Fig. 4C). Similarly, Mcl-1 protein was measured by immunoblotting at serial timepoints, with vinculin loading control (Fig. 4B). Mcl-1 protein turnover was also accelerated in combination-treated, compared with single drug–treated and DMSO-treated Ba/F3-ITD, MV4-11, and MOLM-14 cells, and accelerated turnover was abrogated in cells pretreated with MG-132 (Fig. 4D), consistent with our previous results (12).

### Pim and FLT3 Inhibitor Cotreatment Downregulates c-Myc Through Phosphorylation at T58

**FIGURE 5:**
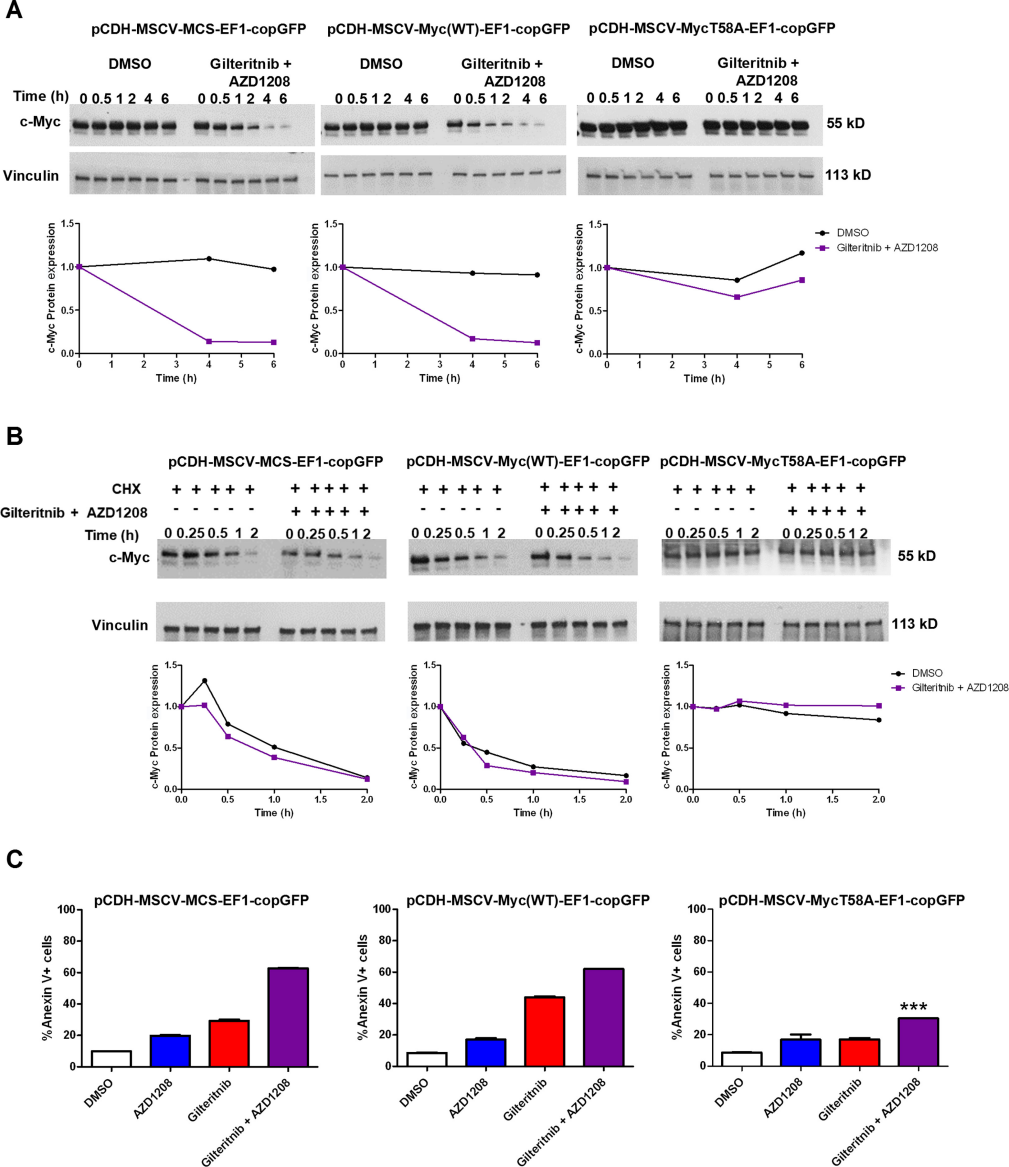
T58A-mutated c-Myc confers resistance to c-Myc downregulation and to apoptosis induction by gilteritinib and AZD1208 combination treatment. **A,** Ba/F3-ITD cells infected with pCDH-MSCV-MycT58A-EF1a-copGFP, containing c-Myc with a mutation changing threonine to alanine at residue 58, preventing phosphorylation, pCDH-MSCV-Myc(WT)-EF1a-copGFP, containing wild-type c-Myc, or pCDH-MSCV-MCS-EF1-copGFP empty vector were treated with either gilteritinib and AZD1208 or DMSO control and serial samples were immunoblotted for c-Myc and vinculin loading control. Densitometric analysis is also shown. **B,** To measure c-Myc protein turnover, cells were pretreated with CHX for 1 hour and then treated with gilteritinib and AZD1208 (+) or DMSO control (−). Serial samples were immunoblotted for c-Myc and vinculin loading control. Densitometry was performed. c-Myc was normalized to vinculin and 50% protein turnover timepoints were determined to be 1.035 versus 1.2 hours for gilteritinib and AZD1208 versus DMSO control for empty vector, 0.6 versus 0.72 for wild-type c-Myc and more than 2 hours for both for T58A c-Myc. **C,** Cells infected with pCDH-MSCV-MycT58A-EF1a-copGFP, pCDH-MSCV-Myc(WT)-EF1a-copGFP or pCDH-MSCV-MCS-EF1-copGFP empty vector were treated with gilteritinib and/or AZD1208, or DMSO control, for 48 hours, and apoptosis was measured. Apoptosis induction by gilteritinib and AZD1208 combination was significantly reduced in cells infected with pMSCVpuro*-*Flag*-*cMyc T58A, compared with empty vector control (***, *P* <0.0001).

To test the role of c-Myc T58 phosphorylation in c-Myc downregulation by gilteritinib and Pim inhibitor cotreatment, Ba/F3-ITD cells infected with pCDH-MSCV-MycT58A-EF1a-copGFP plasmid, containing c-Myc with a mutation changing threonine to alanine at residue 58, preventing phosphorylation, or with pCDH-MSCV-Myc(WT)-EF1a-copGFP plasmid, containing wild-type c-Myc, or pCDH-MSCV-MCS-EF1-copGFP empty vector control, were treated with either gilteritinib and AZD1208 or DMSO control and c-Myc protein expression was measured by immunoblotting at serial timepoints, with vinculin protein loading control. c-Myc protein levels did not decrease with gilteritinib and AZD1208 treatment in cells infected with pCDH-MSCV-MycT58A-EF1a-copGFP plasmid, in contrast to cells infected with pCDH-MSCV-Myc(WT)-EF1a-copGFP plasmid or with pCDH-MSCV-MCS-EF1-copGFP empty vector (Fig. 5A). c-Myc protein turnover was then studied in cells treated with gilteritinib and AZD1208 combination or DMSO control, demonstrating slower c-Myc protein turnover in cells with pCDH-MSCV-MycT58A-EF1a-copGFP, relative to cells with pCDH-MSCV-Myc(WT)-EF1a-copGFP or with empty vector (Fig. 5B). Thus, c-Myc T58 phosphorylation inhibited c-Myc downregulation by gilteritinib and Pim inhibitor cotreatment, and the effect of overexpression of Myc with T58A was due to expression of a non-phosphorylatable variant rather than to a general effect of Myc overexpression.

To determine the role of c-Myc T58 phosphorylation in apoptosis induction by cotreatment, cells infected with pCDH-MSCV-MycT58A-EF1a-copGFP, pCDH-MSCV-Myc(WT)-EF1a-copGFP or pCDH-MSCV-MCS-EF1-copGFP empty vector control were treated with gilteritinib and AZD1208 combination, single drugs or DMSO control, and apoptosis was measured after 48 hours. Apoptosis induction by gilteritinib and AZD1208 combination was significantly reduced in cells infected with pCDH-MSCV-MycT58A-EF1a-copGFP, compared with pCDH-MSCV-Myc(WT)-EF1a-copGFP or pCDH-MSCV-MCS-EF1-copGFP empty vector (Fig. 5C). Apoptosis induction by gilteritinib was also reduced, but to a lesser extent (Fig. 5C). Thus, c-Myc T58 phosphorylation contributes to apoptosis induction by cotreatment.

### FLT3 and Pim Inhibitor Cotreatment Downregulates Mcl-1 Through Phosphorylation at S159

**FIGURE 6:**
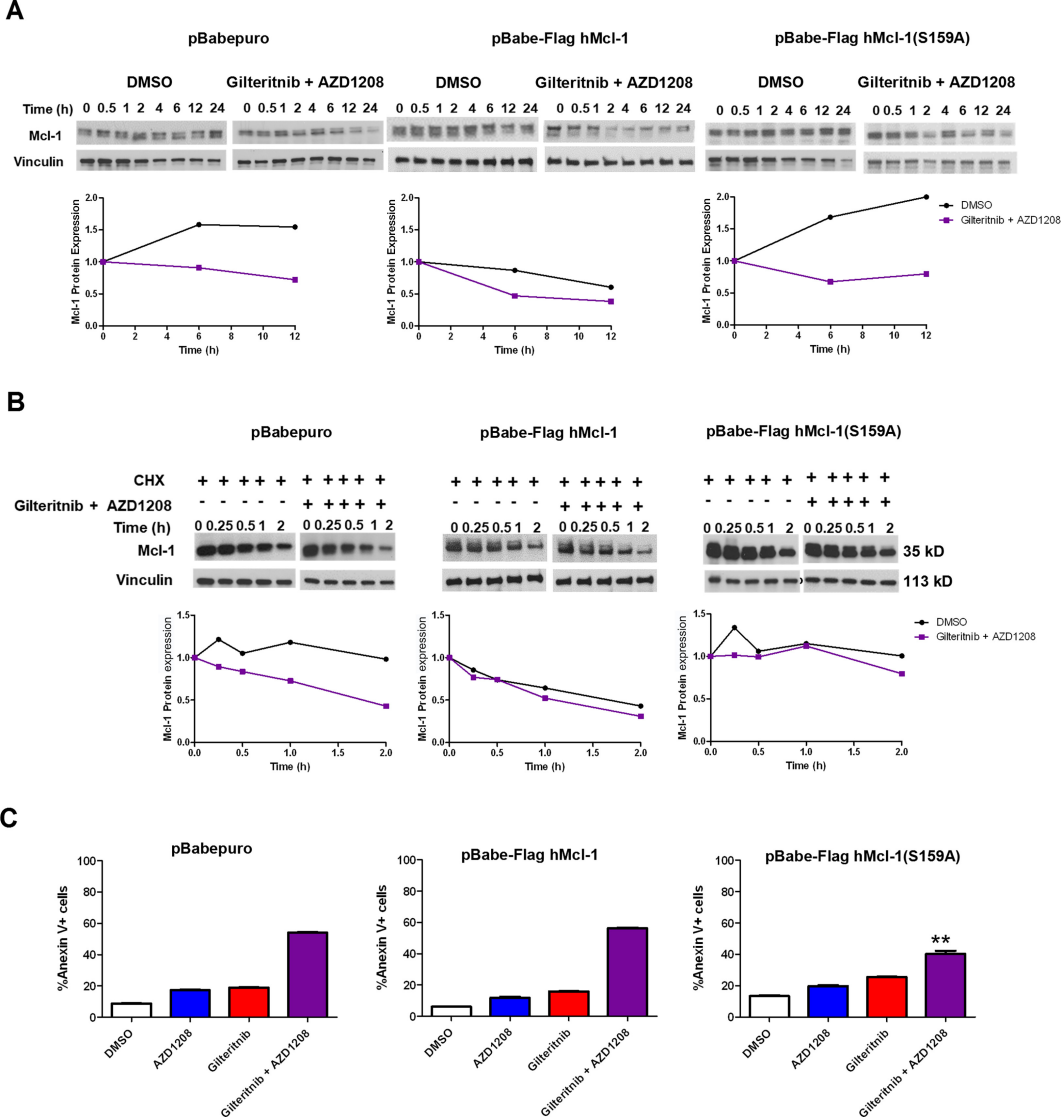
S159A-mutated Mcl-1 confers resistance to Mcl-1 downregulation and to apoptosis induction by gilteritinib and AZD1208 combination treatment. **A,** Ba/F3-ITD cells infected with pBabe-Flag hMcl-1-S159A, containing Mcl-1 with a mutation changing serine to alanine at residue 159, preventing phosphorylation, pBabe-Flag hMcl-1 plasmid, containing wild-type Mcl-1, or pBABE-puro empty vector were treated with either gilteritinib and AZD1208 or DMSO control, and serial samples were immunoblotted for Mcl-1 and vinculin loading control. Densitometric analysis is also shown. **B,** To measure Mcl-1 protein turnover, cells were pretreated with CHX for 1 hour and then treated with gilteritinib and AZD1208 (+) or DMSO control (−). Serial samples were immunoblotted for c-Myc and vinculin loading control. Densitometric analysis was performed. Mcl-1 was normalized to vinculin and 50% protein turnover timepoints were determined to be 1.75 versus more than 2 hours for gilteritinib and AZD1208 versus DMSO control for empty vector, 1.27 versus 1.6 for wild-type Mcl-1 and more than 2 hours for both for S159A Mcl-1. **C,** Cells infected with pBabe-Flag hMcl-1-S159A, pBabe-Flag hMcl-1 or pBABE-puro empty vector control were treated with gilteritinib and/or AZD1208, or DMSO control, for 48 hours, and apoptosis was measured. Apoptosis induction by gilteritinib and AZD1208 combination was significantly reduced in cells infected with pBABE-puroS159A compared with empty vector control (**, *P* < 0.001).

To test the role of S159 phosphorylation in Mcl-1 downregulation by FLT3 and Pim inhibitor cotreatment, Ba/F3-ITD cells transfected with pBabe-Flag hMcl-1-S159A plasmid, containing Mcl-1 with a mutation changing serine to alanine at residue 159, preventing phosphorylation, or with pBabe-Flag hMcl-1 plasmid, containing wild-type Mcl-1 or pBabe-puro empty vector control were treated with gilteritinib and AZD1208 combination, or with DMSO control. Cells infected with pBabe-Flag hMcl-1-S159A plasmid showed less Mcl-1 protein downregulation (Fig. 6A) and reduced Mcl-1 protein turnover (Fig. 6B), compared with cells infected with pBabe-Flag hMcl-1 plasmid or pBabe-puro empty vector. Thus Mcl-1 S159 phosphorylation inhibited Mcl-1 downregulation by gilteritinib and Pim inhibitor cotreatment, and the effect of overexpression of Mcl-1 with S159A was due to expression of a non-phosphorylatable variant rather than to a general effect of Mcl-1 overexpression.

Apoptosis induction by gilteritinib and AZD1208 combination was also significantly reduced in cells infected with pBabe-Flag hMcl-1-S159A compared with pBabe-Flag hMcl-1 plasmid or pBabe-puro empty vector, while apoptosis induction by single drugs did not differ (Fig. 6C). Thus Mcl-1 S159 phosphorylation contributes to apoptosis induction by gilteritinib and AZD1208 combination.

### Pim and FLT3 Inhibitor Cotreatment Activates GSK-3β

**FIGURE 7:**
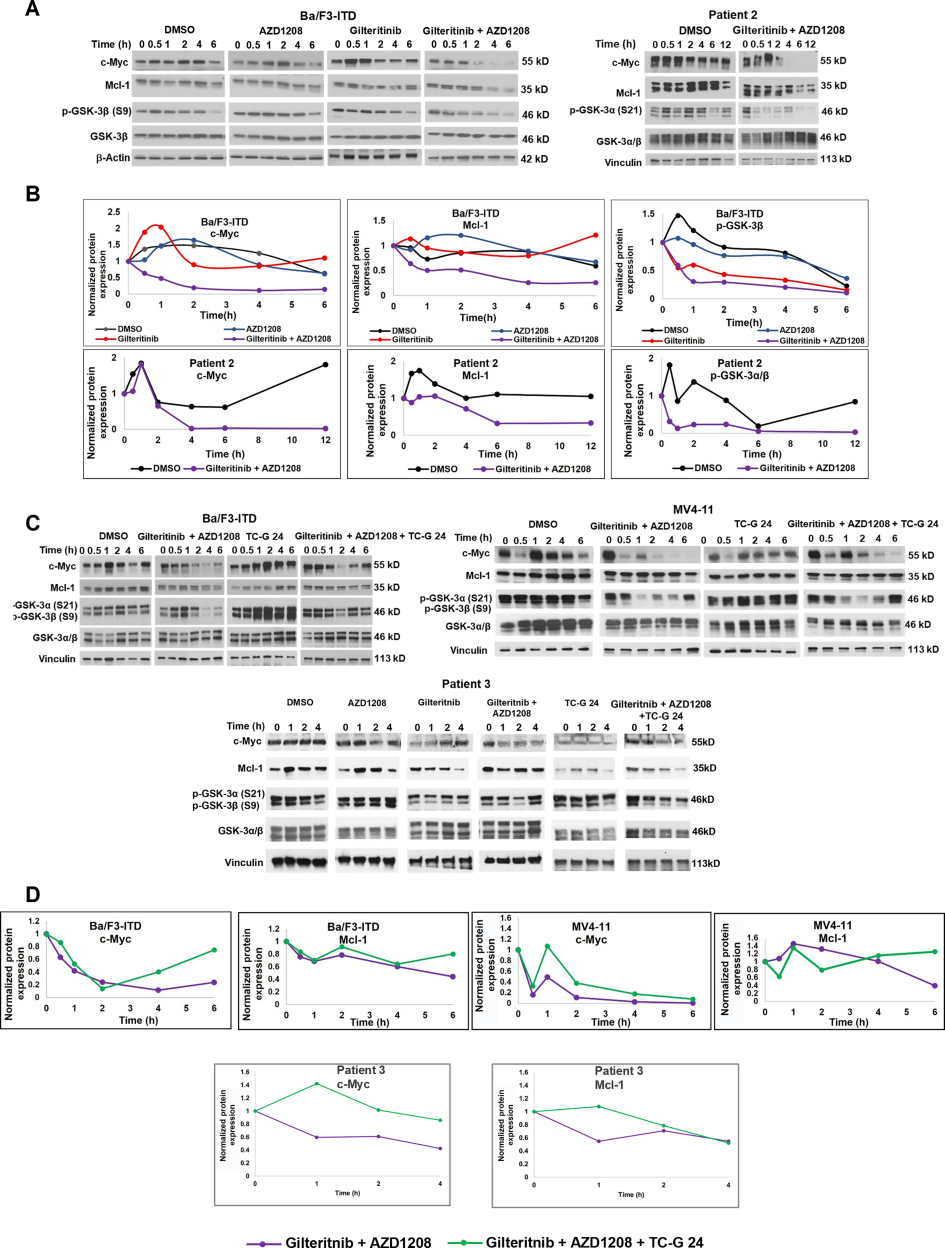
Concurrent Pim and FLT3 inhibitor treatment activates GSK-3β, and GSK-3β inhibitor abrogates c-Myc and Mcl-1 downregulation. **A,** Ba/F3-ITD cells and FLT3-ITD AML patient blasts were treated with gilteritinib and/or AZD1208, or DMSO control, and expression of c-Myc, Mcl-1, p-GSK-3α,β (S9/S21), GSK-3α,β and β-actin or vinculin loading control at serial time points was measured by immunoblotting. Data in **A** are shown graphically in **B**. **C,** Ba/F3-ITD and MV4-11 cells and FLT3-ITD AML patient blasts were treated with gilteritinib and/or AZD1208, or DMSO control, with and without the GSK-3β inhibitor TC G-24 at 20 nM, and expression of c-Myc, Mcl-1, p-GSK-3α,β (S9/S21), GSK-3α,β and β-actin or vinculin loading control was measured at serial time points by immunoblotting. Data in **C** are shown graphically in **D**.

The serine/threonine kinase GSK-3β phosphorylates c-Myc and Mcl-1 at T58 and S159, respectively, to promote their proteasomal degradation. To understand whether gilteritinib and Pim inhibitor cotreatment enhances c-Myc and Mcl-1 proteasomal degradation through GSK-3β activation, p-GSK-3β (inactivated) and GSK-3β expression was measured in Ba/F3-ITD cells and FLT3-ITD AML patient blasts treated with gilteritinib and/or AZD1208, or DMSO control. Cotreatment decreased GSK-3β (S9) phosphorylation in Ba/F3-ITD cells and FLT3-ITD AML blasts, and the decrease in GSK-3β (S9) phosphorylation preceded c-Myc and Mcl-1 downregulation (Fig. 7A and B).

### GSK-3β Inhibition Abrogates c-Myc and Mcl-1 Downregulation by FLT3 and Pim Inhibitor Cotreatment

To determine whether c-Myc and Mcl-1 downregulation is caused by GSK-3β activation, Ba/F3-ITD and MV4-11 cells and FLT3-ITD AML patient blasts were treated with gilteritinib and AZD1208 in the presence and absence of the GSK-3β inhibitor TG-C 24, and c-Myc and Mcl-1 expression was measured by immunoblotting at serial timepoints. TC-G 24 treatment inhibited c-Myc and Mcl-1 downregulation by gilteritinib and AZD1208 cotreatment in Ba/F3-ITD and MV4-11 cells and FLT3-ITD AML primary patient blasts (Fig. 7C and D), demonstrating that c-Myc and Mc-1 downregulation by cotreatment results at least in part from GSK-3β activation.

### Gilteritinib and Pim Inhibitor Combination Rapidly Inactivates AKT, but AKT Inactivation is not Necessary for GSK-3β Activation, c-Myc or Mcl-1 Downregulation or Apoptosis Induction

We recently showed that PP2A-activating drugs enhance FLT3 inhibitor efficacy in cells with FLT3-ITD through AKT inhibition, which activates GSK-3β, resulting in GSK-3β–mediated enhanced c-Myc and Pim-1 proteasomal degradation (24). We therefore studied the possible role of AKT inhibition in posttranslational c-Myc and Mcl-1 downregulation in cells with FLT3-ITD treated with gilteritinib and Pim inhibitor combination.

AZD1208 and gilteritinib combination treatment of Ba/F3-ITD cells rapidly inactivated AKT, decreasing both p-AKT (S473) and (T308) expression, compared with single drugs or DMSO control, while total AKT levels remained unchanged (Supplementary Fig. S2A).

To test whether AKT inactivation is necessary for GSK-3β activation and c-Myc and Mcl-1 downregulation, Ba/F3-ITD cells infected with myr-AKT plasmid, which renders AKT constitutively active, or empty vector control were treated with AZD1208 and/or gilteritinib, or DMSO control. AZD1208 and gilteritinib cotreatment activated GSK3-β and downregulated c-Myc and Mcl-1 (Supplementary Fig. S2B), and induced apoptosis (Supplementary Fig. S2C) similarly in cells infected with myr-AKT or empty vector control. Therefore, while Pim and FLT3 inhibitor combination rapidly inactivates AKT, AKT inactivation is not necessary for GSK3-β activation, c-Myc or Mcl-1 downregulation or apoptosis induction by gilteritinib and AZD1208 combination treatment.

### ERK1/2 is Inactivated Similarly by Gilteritinib with or without AZD1208

To determine differential effects of gilteritinib and Pim inhibitor treatment, relative to gilteritinib treatment, on ERK1/2 inactivation, Ba/F3-ITD cells were treated with gilteritinib and/or AZD1208, or DMSO control, and expression of p-ERK and ERK was measured at serial timepoints by immunoblotting. Both gilteritinib and AZD1208 and gilteritinib alone similarly rapidly inactivated ERK1/2 (Supplementary Fig. S2D).

## Discussion

**FIGURE 8:**
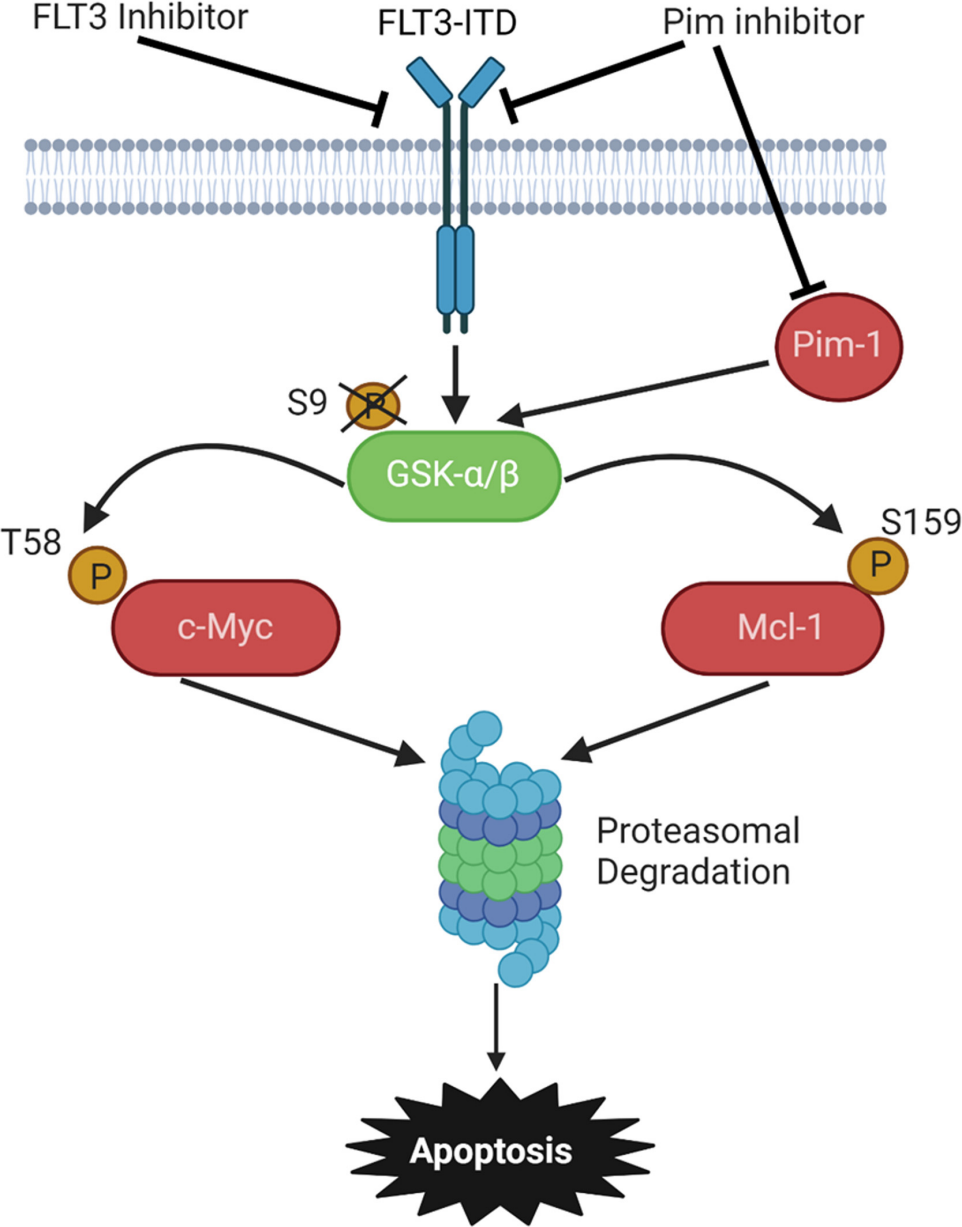
Summary figure. Summary figure showing the proposed effects of combined FLT3 and Pim kinase inhibition in cells with FLT3-ITD.

FLT3-ITD is present in blasts of 30% of patients with AML and is associated with poor treatment outcomes. FLT3 inhibitors have clinical activity, but efficacy is generally limited and transient. We previously showed that concurrent targeting of the serine/threonine kinase Pim-1, which is upregulated downstream of STAT5 in the aberrant FLT3-ITD signaling pathway (6, 7), enhances apoptosis induction by FLT3 inhibitors by posttranslationally downregulating the antiapoptotic protein Mcl-1 through enhanced proteasomal degradation (12). Here we show that concurrent targeting of Pim-1 and FLT3 in cells with FLT3-ITD also causes posttranslational downregulation of c-Myc, preceding downregulation of Mcl-1, and that posttranslational downregulation of both proteins is mediated by activation of GSK-3β, which phosphorylates the two proteins at T58 and S159, respectively, and thereby tags them for proteasomal degradation. This proposed mechanism is illustrated in Fig. 8.

c-Myc is overexpressed in AML with FLT3-ITD through both transcriptional upregulation (7) and posttranslational upregulation by Pim-1, which stabilizes c-Myc protein through increased phosphorylation at S62 and decreased phosphorylation at T58 (19). c-Myc overexpression contributes to leukemogenesis in cells with FLT3-ITD, as demonstrated by marked reduction in cell proliferation following c-Myc siRNA knockdown (7). FLT3 inhibitors, as single agents, downregulate c-Myc (7), which we also showed here. There are no direct c-Myc inhibitors available or in clinical development, and Pim inhibitors have been proposed as indirect c-Myc inhibitors in other malignancies, including prostate and breast cancers (39–41). In our studies of FLT3-ITD–expressing cell lines and AML patient blasts, Pim inhibitors alone did not downregulate c-Myc, but Pim and FLT3 inhibitor cotreatment downregulated c-Myc more markedly and more rapidly than FLT3 inhibitor alone, likely contributing to enhanced apoptosis induction by combination therapy, demonstrated previously (9–12) and here.

The antiapoptotic protein Mcl-1 is also a key target in AML with FLT3-ITD. Mcl-1 inhibition induces apoptosis, while enforced Mcl-1 expression inhibits apoptosis (8). FLT3-ITD–expressing cells express Mcl-1 at high levels, FLT3 inhibition downregulates Mcl-1, and blocking STAT5 activation completely abrogates Mcl-1 expression (8). In addition, suppressing endogenous Mcl-1 by siRNA or flavopiridol treatment sensitizes FLT3-ITD–expressing hematopoietic cells to cytotoxic and targeted therapeutics (42). Direct Mcl-1 inhibitors have been associated with cardiotoxicity (43, 44) and indirect approaches to Mcl-1 inhibition or downregulation (45) appear preferable. We show here and showed previously (12) that FLT3 and Pim inhibitor cotreatment downregulates Mcl-1 in cells with FLT3-ITD, more markedly and more rapidly than FLT3 inhibitors alone.

We found that posttranslational downregulation of both c-Myc and Mcl-1 resulted from activation of GSK-3β, which phosphorylates these proteins at T58 and S159, respectively, thereby tagging them for proteasomal degradation. We previously found that PP2A-activating drugs, which overcome inactivation of the tumor suppressor PP2A in cells with FLT3-ITD, also enhance efficacy of FLT3 inhibitors through activation of GSK-3β, which phosphorylates c-Myc and Pim-1, increasing posttranslational downregulation of both proteins and enhancing apoptosis (24). We did not study Mcl-1, but it is likely also downregulated.

FLT3 and Pim inhibitor cotreatment was not AKT-dependent or ERK-dependent, and enhanced efficacy thus likely occurred entirely through the STAT5 pathway. GSK-3 is a substrate of AKT in the (PI3K)-Akt-mTOR pathway (21). In our work here, gilteritinib and Pim inhibitor cotreatment inhibited AKT inhibition and activated GSK-3β, but GSK-3β activation was independent of AKT inhibition, as it occurred in cells with (constitutively activated) myr-AKT. Moreover, AZD1208 and gilteritinib cotreatment downregulated c-Myc and Mcl-1 and induced apoptosis similarly in cells infected with myr-AKT and with empty vector. In contrast, we previously showed that PP2A-activating drug and FLT3 inhibitor cotreatment rapidly inactivated AKT in FLT3-ITD–expressing cells through dephosphorylation at both S473 and T308, and that AKT inactivation caused GSK-3β activation. In addition, PP2A-activating drug and FLT3 inhibitor cotreatment caused GSK-3β activation in parental Ba/F3-ITD cells and Ba/F3-ITD cells infected with empty vector, but not with myr-AKT, and treatment with the AKT inhibitor MK-2206 caused GSK-3β activation (24). GSK-3β activation has also been reported to be ERK dependent (46), but ERK was similarly inactivated by gilteritinib and AZD1208 and by gilteritinib alone. As above, enhanced efficacy of Pim and FLT3 inhibitor treatment thus likely occurred entirely through enhanced effects on the STAT5 pathway (9).

The two GSK-3 paralogs, GSK-3α and GSK-3β, have variably been reported to have tumor suppressor and oncogenic properties in different acute leukemia types and subtypes (47, 48). GSK-3α or GSK-3β was found to be necessary for survival and proliferation of cells with *KMT2A* (*MLL*) gene rearrangements *in vitro* and *in vivo*, through destabilization of p27^Kip1^, a cyclin-dependent kinase inhibitor that is a tumor suppressor protein (49). Treatment with selective GSK-3 inhibitors, knockdown of GSK-3α or genetic ablation of GSK-3β inhibited proliferation of cells with *KMT2A* rearrangements, and this antiproliferative effect was inhibited by p27Kip1 knockdown (49). In contrast, GSK-3 inhibition did not produce antiproliferative effects and did not increase expression of p27Kip1 in acute leukemia cells with other gene rearrangements, including *TEL*-*AML1*, *E2A*-*HLF,* and *E2A*-*PBX1* (49). Of note, GSK-3 inhibitors have also exhibited clinical activity in some patients with refractory solid tumors, and the roles of GSK-3 and consequences of its modulation would seem to be contextual and tumor specific (50).

Prognostic significance of both GSK-3 phosphorylation and localization has also been reported in AML. Lower levels of p-GSK-3α/β in AML cells, measured by reverse phase protein analysis, correlated with longer remission duration and overall survival in patients with intermediate-risk, but not unfavorable-risk, karyotypes (51). Notably, in patients with intermediate-risk karyotypes and FLT3 mutations, lower p-GSK-3α/β levels, indicating GSK-3α/β activation, strongly correlated with longer remission duration (51). Aberrant nuclear localization of GSK-3β has also been described in AML, correlating with AML growth *in vitro* and *in vivo*, drug (daunorubicin and cytarabine) resistance and shorter patient survival (52). A proposed mechanism is promotion of nuclear localization of the NFκB subunit p65 by nuclear GSK-3β, increasing transcription of NFκB target genes (52).

Both our work here and previous work (24, 53) support GSK-3β activation as a therapeutic strategy in AML with FLT3-ITD. Here and previously (24), we found that GSK-3 activation destabilized c-Myc, Pim-1, and Mcl-1 and enhanced apoptosis induction in cells with FLT3-ITD. In other work, GSK-3α and downstream SPRY3 were found to be markedly downregulated in quizartinib-resistant FLT3-ITD–expressing AML cells, associated with restored RAS/MAPK or Wnt signaling, and knockout of GSK-3α and GSK-3β or SPRY3 conferred resistance to quizartinib (53). Most recently FLT3 inhbitors, including type I (midostaurin, crenolanib, and gilteritinib) and type II (quizartinib and sorafenib) at concentrations (50 nmol/L) that induced apoptosis in MV4-11 and MOLM-13 cells, with FLT3-ITD, were found to decrease levels of p-ERK, p-AKT, and p-GSK-3β and to downregulate Mcl-1 and Bim (54). Moreover, the GSK-3β inhibitors SB216763 and CHIR-99021 inhibited apoptosis induction and Mcl-1 downregulation, but not Bim induction (54).

In chronic myeloid leukemia (CML), which is driven by the oncogenic tyrosine kinase BCR-ABL, GSK-3 was reported to be inactivated and enforced expression of constitutively active GSK-3 reduced proliferation and potentiated BCR-ABL inhibitor–induced apoptosis in both BCR-ABL inhibitor–sensitive and -resistant cells (55). This work suggested therapeutic efficacy of GSK-3 activation in CML. Indeed, the Pim-1 kinase inhibitor SMI-4a was subsequently found to exert antitumor effects in both imatinib-sensitive and -resistant CML cells by increasing GSK-3β activity (56).

GSK-3β activation has also been described in response of colorectal cancer cells to kinase inhibitors. In one study, treatment with the multi-kinase inhibitor sorafenib inactivated ERK1/2 by dephosphorylation at T202/Y204, resulting in GSK-3β activation by dephosphorylation at S9, promoting p65 phosphorylation and expression of the proapoptotic Bcl-2 protein family member PUMA (57). In a subsequent study, treatment of colorectal cancer cells with gilteritinib, which inhibits AXL as well as FLT3, caused AKT inhibition and consequent GSK-3β activation, which resulted in nuclear translocation of p65 and induction of PUMA as a mechanism of apoptosis induction, and GSK-3β knockdown suppressed gilteritinib-induced p65 phosphorylation and induction of PUMA (58). The same mechanism was demonstrated for the multi-kinase inhibitor regorafenib in colorectal cancer cells (59).

On the basis of our work here and our previous work (24), GSK-3β  activation appears to be an effective strategy for optimizing response to FLT3 inhibitors, through posttranslational downregulation of c-Myc, Mcl-1, and Pim-1, key proteins driving proliferation and resistance to apoptosis in AML cells with FLT3-ITD. This pathway also appears to be relevant to other tyrosine kinase–driven leukemias, as well as solid tumors.

Finally, our data provide support for potential development of a clinical trial combining TP-3654 and gilteritinib in AML with FLT3-ITD, with correlative laboratory studies. Gilteritinib is FDA approved for treatment of relapsed and refractory FLT3-mutated AML (29). AZD1208 was generally well tolerated in a phase I trial in patients with AML and solid tumors in two dose-escalation studies, but it increased CYP3A4 activity after multiple dosing, resulting in increased drug clearance, and was therefore withdrawn from clinical studies (60). In contrast, TP-3654 has been well tolerated in clinical trials (37, 38) and remains in active clinical development. As an additional consideration, because Pim and FLT3 inhibitor combination treatment enhances Mcl-1 downregulation, relative to FLT3 inhibitor alone, and Mcl-1 upregulation is a mechanism of resistance to the Bcl-2 inhibitor venetoclax (61), *in vitro*, *in vivo* and potentially clinical exploration of TP-3654 with giletritinib and venetoclax (62, 36) as triple combination therapy would be of interest.

## Supplementary Material

Supplementary Table S1 Clinical information on patients with AML with FLT3-ITD whose samples were studied.

Supplementary Figure S1 Apoptosis dot plots

Supplementary Figure S2 Concurrent Pim and FLT3 inhibitor treatment inhibits AKT, but GSK-3β activation, c-Myc and Mcl-1 downregulation and apoptosis induction are independent of AKT inhibition as well as ERK inhibition.
