# Supplementary material for: Pim Kinase Inhibitors Increase Gilteritinib Cytotoxicity in FLT3-ITD Acute Myeloid Leukemia Through GSK-3β Activation and c-Myc and Mcl-1 Proteasomal Degradation
Source: Cancer Res Commun. 2024 Feb 16;4(2):431–45. doi: 10.1158/2767-9764.CRC-23-0379 (PMC10870818; doi:10.1158/2767-9764.CRC-23-0379)
Supplement: Supplementary Table S1 — Clinical information on patients with AML with FLT3-ITD whose samples were studied. [file crc-23-0379-s01.docx]

**Supplementary Table S1.** Clinical information on patients with AML with FLT3-ITD whose samples were studied

| **Patient** | **Age/**  **sex** | **Disease**  **status** | **WBC**  **(x10^9^/L)** | **Blasts**  **(%)** | **Karyotype** | **FLT3-ITD**  **size**  **(base pairs)** | **ITD insertion length**  **(base pairs)** | **FLT3-ITD**  **allelic**  **burden (%)** |
| --- | --- | --- | --- | --- | --- | --- | --- | --- |
| 1 | 79M | Diagnosis | 160.3 | 98 | 46,XY | 350; 379 | 22; 51 | 58; 24 |
| 2 | 85F | Relapse | 32.2 | 45 | 46,XX | 356 | 28 | 52 |
| 3 | 63M | Diagnosis | 104.1 | 40 | 46,XY | 370 | 42 | 33 |
