# Supplementary material for: Pim Kinase Inhibitors Increase Gilteritinib Cytotoxicity in FLT3-ITD Acute Myeloid Leukemia Through GSK-3β Activation and c-Myc and Mcl-1 Proteasomal Degradation
Source: Cancer Res Commun. 2024 Feb 16;4(2):431–45. doi: 10.1158/2767-9764.CRC-23-0379 (PMC10870818; doi:10.1158/2767-9764.CRC-23-0379)
Supplement: Supplementary Figure S1 — Apoptosis dot plots [file crc-23-0379-s02.docx]

**Supplementary Figure S1. Apoptosis dot plots.** MV4-11 and MOLM-14 cells were treated with the FLT3 inhibitor gilteritinib (10 nM) and/or the Pim inhibitor AZD1208 (1 µM) or TP-3654 (1 µM), or DMSO control, for 48 hours in triplicate experiments**.** Apoptosis was analyzed by Annexin V and PI staining, measured by flow cytometry. Dot plots from duplicate experiments are shown.

**
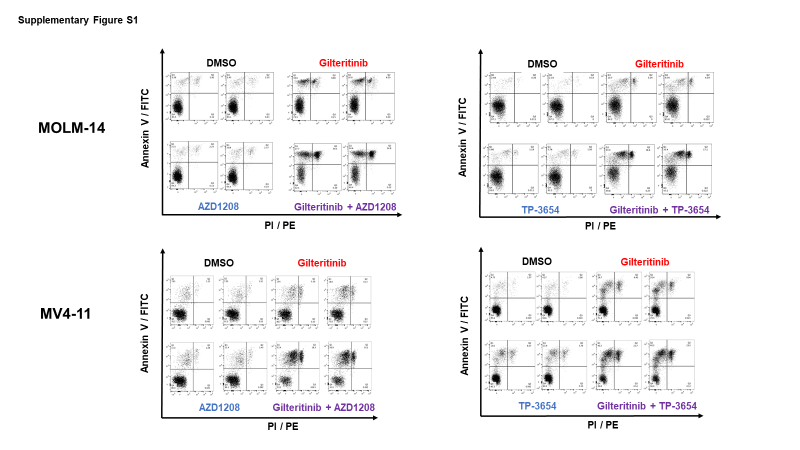
**
