# Supplementary material for: Pim Kinase Inhibitors Increase Gilteritinib Cytotoxicity in FLT3-ITD Acute Myeloid Leukemia Through GSK-3β Activation and c-Myc and Mcl-1 Proteasomal Degradation
Source: Cancer Res Commun. 2024 Feb 16;4(2):431–45. doi: 10.1158/2767-9764.CRC-23-0379 (PMC10870818; doi:10.1158/2767-9764.CRC-23-0379)
Supplement: Supplementary Figure S2 — Concurrent Pim and FLT3 inhibitor treatment inhibits AKT, but GSK-3β activation, c-Myc and Mcl-1 downregulation and apoptosis induction are independent of AKT inhibition as well as ERK inhibition. [file crc-23-0379-s03.docx]

**Supplementary Figure S2. Concurrent Pim and FLT3 inhibitor treatment inhibits AKT, but GSK-3β activation, c-Myc and Mcl-1 downregulation and apoptosis induction are independent of AKT inhibition as well as ERK inhibition. A.** Ba/F3-ITD cells were treated with gilteritinib and/or AZD1208, or DMSO control, and c-Myc, p-AKT (S473 and T308), total AKT and vinculin protein levels were measured in serial samples by immunoblotting. **B.** Ba/F3-ITD cells infected with p-Babe-Puro-Myr-Flag-AKT1, with myristoylated (constitutively active) AKT, or pBabe-Puro empty vector control were treated with AZD1208 and/or gilteritinib, or DMSO control, and c-Myc, p-AKT (S473), AKT, p-GSK-3α,β (S9/S21), GSK-3α,β, Mcl-1 and β-actin or vinculin loading control protein levels were measured in serial samples by immunoblotting. **C.** Apoptosis was measured in parental Ba/F3-ITD cells and Ba/F3-ITD cells infected with p-Babe-Puro-Myr-Flag-AKT1 or pBabe-Puro empty vector control treated with AZD1208 and/or gilteritinib, or DMSO control, for 48 hours. Apoptosis induction was similar. **D.** Ba/F3-ITD cells were treated with gilteritinib and/or AZD1208, or DMSO control, and expression of p-ERK1/2 and ERK1/2 was measured at serial time points by immunoblotting. p-ERK1/2 was similarly rapidly downregulated by gilteritinib alone and by gilteritinib and AZD1208.

**
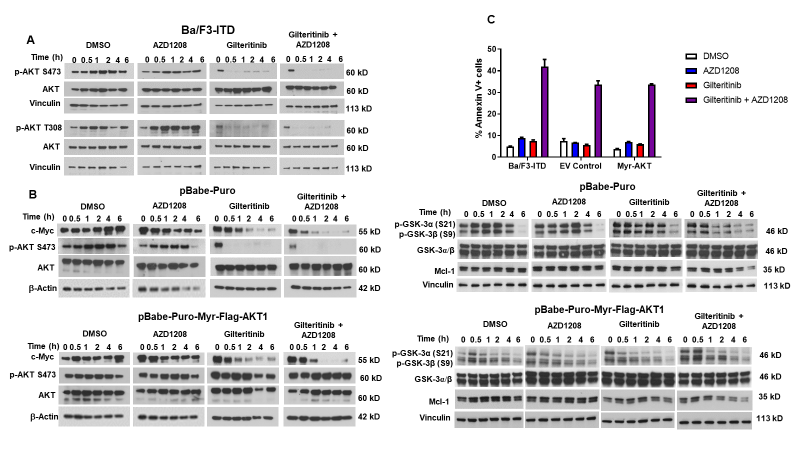
**
